# The Value of Gude’s Pepto-Mangan in Anemia

**Published:** 1903-01

**Authors:** 


### ABSTRACTS.

### The Value of Gude's Pepto-mangan in Anemia.

Enrique Diago and José F. Benitez, both of Havana, in the *Progreso Medico*, April, 1902, say: anemia is a very common disease in Cuba and consequently one against which the physician is often obliged to contend in the practice of his art. While the use of the ordinary iron preparations often give all the effects that could be desired, yet they frequently produce a condition which may be regarded as a secondary disease—constipation. In looking about for a preparation which would not present this very serious disadvantage, which cannot always be counteracted by the coincident administration of laxatives, we came across Gude's pepto-mangan, which seemed a remedy worth a trial in a large series of cases. Accordingly, we began to treat all our cases of anemia, in which iron was indicated, with Gude's pepto-mangan. Our expectations were more than realised, when we noted its efficiency in combating the condition, and its perfect palatability and freedom from constipating after-effects.

One of the reporters, Dr. Benitez, chief of the laboratory of the hospital, kept minute records of all the cases observed, including a record of the amount of hemoglobin and of the number of the red blood-cells, both before and after treatment. Six cases are included in the statement from which we extract the following two as illustrations of the successful employment of the remedy:

Case I.—N.G., aged 26 years, was admitted to the hospital, suffering from loss of nutrition, emaciation, pallor of the skin and mucous membranes, loss of memory, anorexia, mental depression,—in a word, from all the typical symptoms of anemia. This condition was traced in his case to a chronic malaria, from which the patient had been suffering for a long time. The patient weighed only 102 pounds at the time of admission.

Pepto-Mangan (Gude) was given in doses of two tablespoonfuls twice daily at breakfast and at dinner respectively, with some cinchona wine. The first blood examination showed 2,400,000 red blood corpuscles c.m., by the Thoma-Zeiss method. Ten days after the beginning of the treatment, this patient, who had been so extremely pale when he entered, began to improve as regards the color of his cheeks and general appearance. His general wellbeing was so marked that he spoke with pleasure of the great improvement in his condition which had taken place since he had been taking the new remedy at our hospital. In these ten days he had gained five pounds in weight and was able to walk around the ward without the lassitude which he had felt when he was admitted. The blood was examined a second time, showing an increase of 300,000 red blood cells. The patient was discharged cured after fifty days' treatment, weighing 130 pounds and with a blood-count indicating 2,800,000 red blood cells c.m.

Case II.—Mrs. C. D., aged 34 years, who gave a history of miscarriage, was admitted with the symptoms of anemia, secondary to the loss of blood occasioned by the accident mentioned. The chief symptoms were emaciation, loss of strength, and gastrointestinal disturbances. She weighed only 90 pounds when she entered the hospital, and her blood showed a marked diminution in the amount of hemoglobin, and only 2,300,000 red blood-cells to the cubic millimetre.

Gude's Pepto-Mangan was prescribed in the same doses as in the preceding case, and all went well until the tenth day, when the patient of her own accord, in order to facilitate the cure, and to accelerate the recovery, took five tablespoonfuls of the preparation during the day, causing a slight disorder of the stomach. The administration of pepto-mangan was thereupon discontinued, and tablets of bismuth and salol, together with a purgative were given. Five days later, the pepto-mangan was resumed, at first in doses of two teaspoonfuls, and two days later in doses of two tablespoonfuls. The further course of the treatment went on without any mishap, and the patient recovered completely. On leaving the hospital the hemoglobin was found normal, and the number of red blood cells was found to have increased to 3,500,000 c.m., while the patient's weight had increased twenty-one pounds within fifty days.

These authors conclude their report as follows: to sum up the results obtained with the employment of pepto-mangan (Gude) in the treatment of anemias, we may say conscientiously, that it is the best remedy we know of for this purpose, and that we do not hesitate to commend it to the medical profession at large, and especially to our confrères in Cuba, as an iron preparation that possesses all the advantages that can be demanded of such a remedy and none of the disadvantages that are characteristic of other iron preparations. We would especially emphasise also that pepto-mangan (Gude) is very pleasant to the taste, and is most easily taken by patients of all ages and with the most delicate digestions.

